# A meta-analysis of Hashimoto’s thyroiditis and papillary thyroid carcinoma risk

**DOI:** 10.18632/oncotarget.18620

**Published:** 2017-06-27

**Authors:** Xingjian Lai, Yu Xia, Bo Zhang, Jianchu Li, Yuxin Jiang

**Affiliations:** ^1^ Department of Ultrasound, Chinese Academy of Medical Sciences & Peking Union Medical College Hospital, Beijing, China

**Keywords:** Hashimoto’s thyroiditis, chronic lymphocytic thyroiditis, papillary thyroid carcinoma, thyroid cancer, meta-analysis

## Abstract

**Objective:**

It remains inconclusive whether Hashimoto’s thyroiditis (HT) predisposes patients to the development of papillary thyroid carcinoma (PTC). We conducted a meta-analysis of the available data to address this question.

**Results:**

Twenty-seven eligible studies were selected, including 18 archival thyroidectomy studies, 6 fine-needle aspiration (FNA) studies, and 3 selective FNA or thyroidectomy studies. A total of 76,281 patients, including 12,476 cases of thyroid cancer, were included in these studies. The mean rate of PTC among patients with HT ranged from 1.12% (selective FNA or thyroidectomy studies) to 40.11% (thyroidectomy studies). All three types of studies supported the correlation between HT and PTC. The overall pooled odds ratio (OR) of the PTC risk for HT (HT versus non-HT) was 2.12 (95% confidence interval [CI]: 1.78-2.52).

**Methods:**

We searched all relevant published studies using the citation databases PubMed and Embase. The ORs and corresponding 95% CIs were calculated by the random-effects model for the association between HT and PTC.

**Conclusions:**

Our meta-analysis confirmed that HT predisposed patients to the development of PTC.

## INTRODUCTION

Hashimoto’s thyroiditis (HT) (or chronic lymphocytic thyroiditis) is the most common autoimmune thyroid disease which causes the immune system to attack and destroy the thyroid gland [1]. Papillary thyroid carcinoma (PTC) is the most prevalent thyroid cancer and it represents 80-90% of all thyroid cancers. Dailey et al. were the first to propose an association between HT and PTC in 1955, linking chronic inflammation to neoplastic changes [2]. However, whether HT predisposes patients to the development of PTC is a continuing debate. Some studies have revealed a higher risk of PTC in patients with HT [3–20], while others did not demonstrate the increased risk [21–23]. Lee et al. published a meta-analysis and found that PTC is significantly associated with pathologically confirmed HT [24]. However, Jankovic et al. found that there was no statistically significant correlation between HT and PTC based on 8 fine-needle aspiration (FNA) studies, and 8 thyroidectomy studies, which reported a statistically significant positive correlation, were subject to selection bias [25]. Because inconsistent results have been reported regarding the association between HT and PTC risk, and because many other related studies have been published in recent years, we performed a comprehensive meta-analysis to investigate the possible associations of HT and PTC.

## RESULTS

### Study characteristics

**Figure 1:**
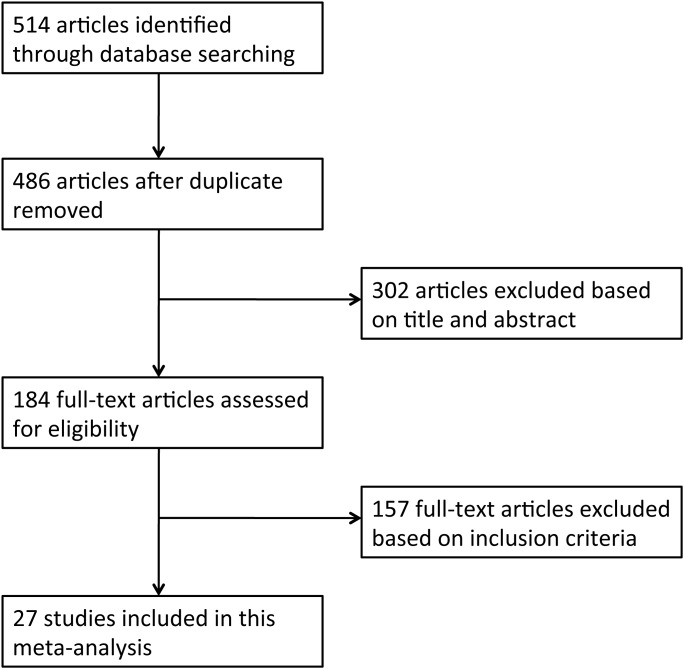
Flow diagram of article selection for this meta-analysis

The literature search strategy identified 514 potentially eligible reports. Finally, 27 studies met the inclusion criteria, including 5 cohort studies [6, 11, 16, 21, 26] and 22 case-control studies [3–5, 7–10, 12–15, 17–20, 22, 23, 27–32] (Figure 1). The detailed characteristics of these studies are shown in Table 1. Eleven studies were conducted in Europe, 9 in Asia, and 7 in the United States. Twenty-four studies evaluated the association between HT and PTC, and 3 studies evaluated the associations between HT and thyroid cancer. A total of 76,281 patients, including 12,476 cases of thyroid cancer, were included in these studies. The quality scores ranged from 5 to 8 with a median of 6 for methodological assessment.

**Table 1:** Characteristics of the studies included in the meta-analysis

Study	Country	Period of enrolment	Study design	Study method	Tumor type	Sample size	No. of PTCs	PTC in HT, %
Chen 2013 [6]	Taiwan	1998-2010	cohort	Selective FNA	TC	7605	19	0.92
Isik 2010 [22]	Turkey	2005-2009	case-control	Selective FNA	TC	500	5	0.67
Mukasa 2011 [16]	Japan	2006	cohort	Selective FNA	PTC	3688	52	1.77
Anil 2010 [21]	Turkey	2006-2009	cohort	FNA	PTC	715	21	1.22
Azizi 2014 [4]	USA	2010-2013	case-control	FNA	TC	2023	233	20.87
de Alcantara-Jones 2015 [28]	Brazil	2011	case-control	FNA	PTC	1049	33	5.20
Fiore 2011 [8]	Italy	2004-2009	case-control	FNA	PTC	9824	659	9.41
Holm 1985 [26]	Sweden	1959-1978	cohort	FNA	PTC	1658	2	0.12
Matesa-Anic 2009 [23]	Croatia	1995-2006	case-control	FNA	PTC	10508	269	1.95
Ahn 2011 [3]	Korea	2000-2005	case-control	thyroidectomy	PTC	303	269	96.67
Bradly 2009 [5]	USA	2000-2008	case-control	thyroidectomy	PTC	678	81	28.38
Buyukasik 2011 [27]	Turkey	1999-2006	case-control	thyroidectomy	PTC	917	61	11.69
Consorti 2010 [7]	Italy	1995-2008	case-control	thyroidectomy	PTC	404	101	36.23
Gul 2010 [9]	Turkey	2005-2008	case-control	thyroidectomy	PTC	613	193	45.65
Kim 2011 [10]	Korea	2003-2007	case-control	thyroidectomy	PTC	1277	1028	92.75
Konturek 2013 [11]	Poland	2002-2010	cohort	thyroidectomy	PTC	7545	636	23.45
Kurukahvecioglu 2007 [12]	Turkey	2001-2005	case-control	thyroidectomy	PTC	922	199	36.63
Larson 2007 [13]	USA	1987-2002	case-control	thyroidectomy	PTC	812	179	34.69
Liu 2014 [14]	China	2008-2013	case-control	thyroidectomy	PTC	6432	1722	43.75
Lun 2013 [15]	China	2004-2012	case-control	thyroidectomy	PTC	2478	676	49.61
Mazokopakis 2010 [29]	Greece	2005-2009	case-control	thyroidectomy	PTC	140	32	28.57
McLeod 1988 [30]	USA	1975-1985	case-control	thyroidectomy	PTC	793	143	23.44
Paparodis 2014 [17]	USA	1994-2013	case-control	thyroidectomy	PTC	2733	893	44.16
Repplinger 2008 [31]	USA	1994-2007	case-control	thyroidectomy	PTC	1198	293	29.03
Siriweera 2010 [18]	Sri Lanka	Not specified	case-control	thyroidectomy	PTC	5357	145	9.46
Zhang 2012 [19]	China	2008-2010	case-control	thyroidectomy	PTC	6109	2797	58.35
Zhang 2014 [20]	China	2004-2011	case-control	thyroidectomy	PTC	8524	1735	29.44

No., number; PTC, papillary thyroid carcinoma; HT, Hashimoto’s thyroiditis; FNA, fine-needle aspiration; TC, thyroid cancer.

Three types of studies were analyzed in this meta-analysis. The first type consists of studies of archival thyroidectomy specimens that were analyzed for the coexistence of HT and PTC. In the second type of study, all patients underwent FNA. However, only selected patients with results suspicious for malignancy underwent thyroidectomy. In the third type of study, only selected patients with suspicious thyroid nodules underwent FNA or thyroidectomy.

### Rate of PTC in patients with HT

**Figure 2:**
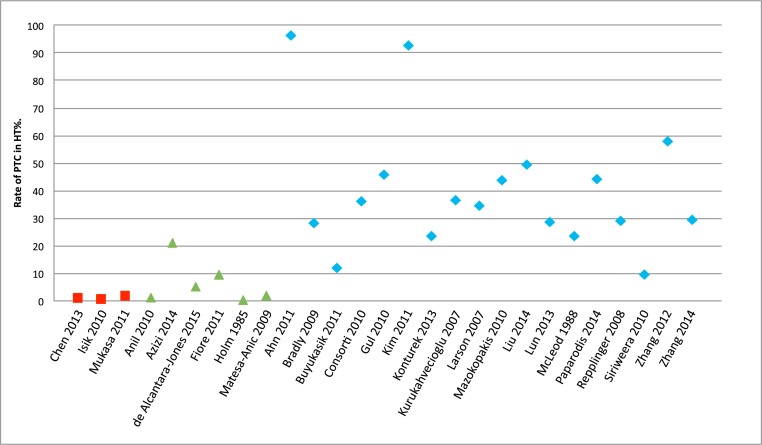
Rate of PTC among patients with HT HT, Hashimoto’s thyroiditis; PTC, papillary thyroid carcinoma. Note: The red squares indicate selective fine-needle aspiration or thyroidectomy studies, the green triangles indicate fine-needle aspiration studies, and the blue rhombuses indicate thyroidectomy studies.

The rate of PTC in patients with HT from the 18 archival thyroidectomy studies (47,235 patients) ranged from 9.46% to 96.67%, with a mean rate of 40.11% (Table 1, Figure 2). Two Korean studies revealed a significantly higher rate than the other thyroidectomy studies (96.67% and 92.75%) [3, 10]. If those studies are excluded as possible outliers, the average rate in the archival thyroidectomy group would be 33.28%.

The rate of PTC among patients with HT from the 6 FNA studies (25,777 patients) ranged from 0.12% to 20.87%, with a mean rate of 6.46%. Azizi et al. found a significantly higher rate than that in the other FNA studies (20.87%) [4]. If that study is excluded as a possible outlier, the average prevalence rate in the FNA group would be 3.58%.

The rate of PTC among patients with HT from the 3 selective FNA or thyroidectomy studies (11,793 patients) ranged from 0.67% to 1.77%, with a mean rate of 1.12%.

### OR of PTC in patients with HT

**Figure 3:**
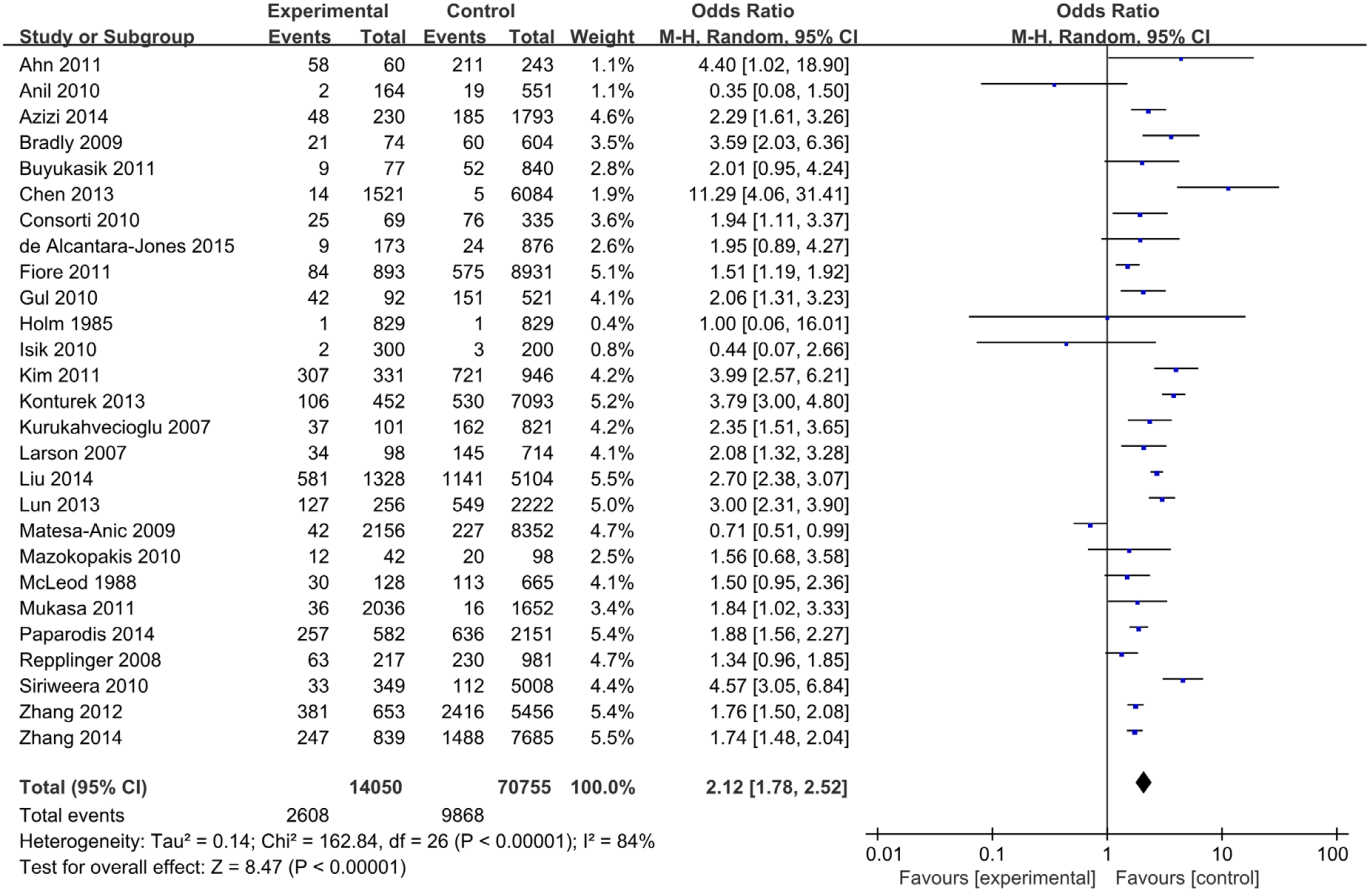
Pooled risk estimates of HT for PTC risk (HT versus non-HT) HT, Hashimoto’s thyroiditis; PTC, papillary thyroid carcinoma.

Figure 3 shows study-specific data, including the odds ratio (OR) and corresponding 95% confidence interval (CI) of the PTC risk for HT (HT versus non-HT). The pooled OR, based on overall studies, was 2.12 (95% CI: 1.78-2.52). However, there was significant heterogeneity among the studies (I^2^ = 84%, P < 0.00001). The corresponding estimates were 2.46 (95% CI: 1.07-5.66) for the cohort studies and 2.03 (95% CI: 1.72-2.41) for the case-control studies. However, there were significant heterogeneities among the cohort studies (I^2^ = 81%, P = 0.0003) and among the case-control studies (I^2^ = 83%, P < 0.00001).

**Figure 4:**
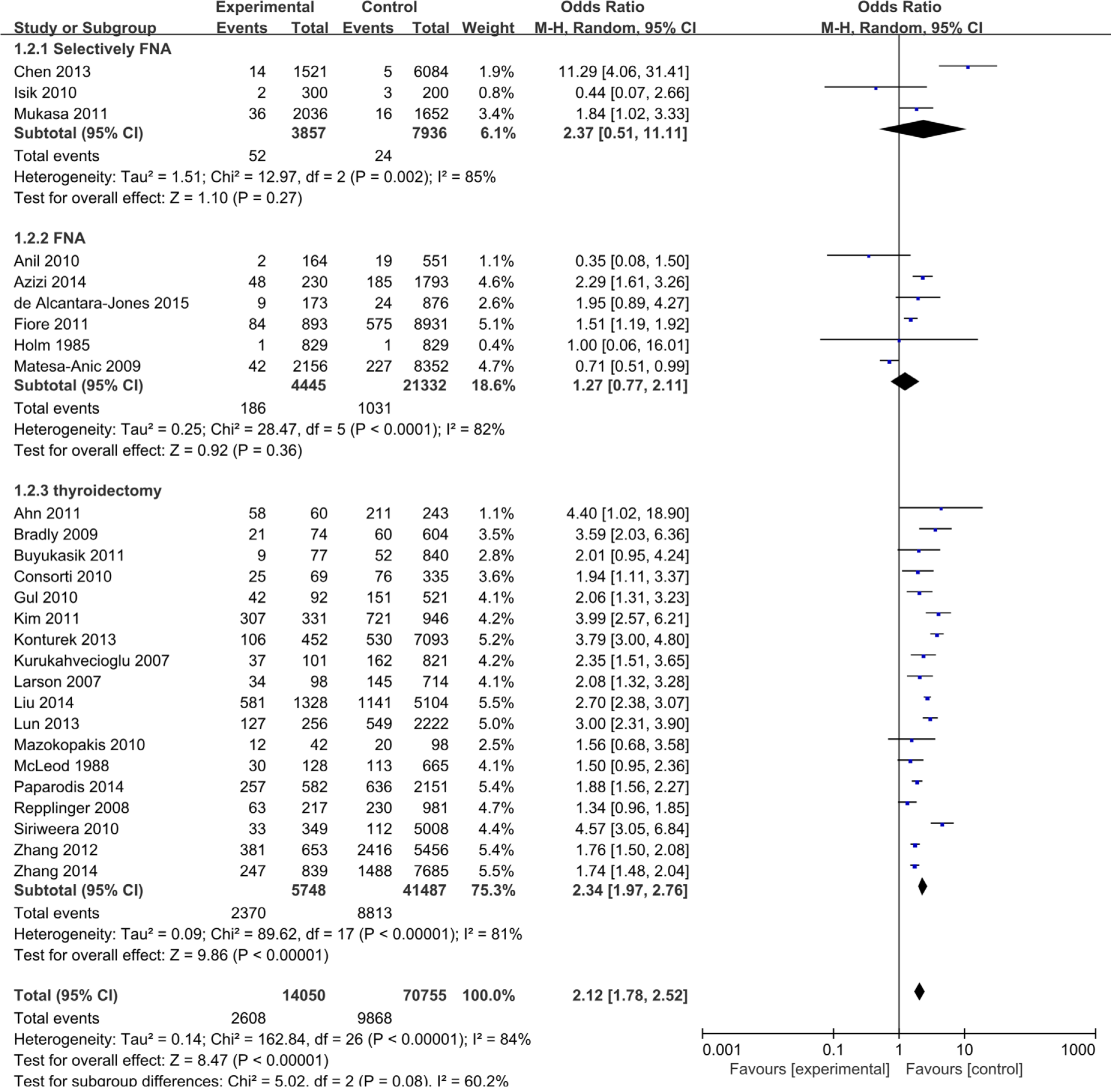
Pooled risk estimates of HT for PTC risk (HT versus non-HT) according to the strata of study method HT, Hashimoto’s thyroiditis; PTC, papillary thyroid carcinoma; FNA, fine needle aspiration

Figure 4 shows a specific study method and the OR and 95% CI of thyroid cancer risk for HT (HT versus non-HT). The OR of PTC in patients with HT in the archival thyroidectomy group (18 studies) ranged from 1.34 to 4.40, with an average OR of 2.34 for a total of 47,271 studied patients (I^2^ = 81%, P < 0.00001). The OR of PTC in patients with HT in the FNA group (6 studies) ranged from 0.35 to 2.29, with an average OR of 1.27 for a total of 25,777 studied patients (I^2^ = 82%, P < 0.0001). The OR of PTC in patients with HT in the selective FNA group (3 studies) ranged from 0.44 to 11.29, with an average OR of 2.37 for a total of 11,793 studied patients (I^2^ = 85%, P = 0.002).

Table 2 presents the results of subgroup analyses regarding the association between HT and PTC risk. Considering the geographical area, the pooled OR for PTC risk was 1.56 (95% CI: 1.01-2.40) in European studies, 2.79 (95% CI: 2.15-3.61) in Asian studies, and 1.92 (95% CI: 1.56-2.35) in American studies. However, significant heterogeneity was found within the geographic areas (all P-values were < 0.10).

**Table 2:** Pooled risk estimates of HT for PTC risk (HT versus non-HT) according to the strata of selected covariates

	No. of studies	OR (95% CI)	P for heterogeneity
Overall	27	2.12 (1.78-2.52)	
Study design			
Cohort	5	2.46 (1.07-5.66)	0.0003
Case-control	22	2.03 (1.72-2.41)	< 0.00001
Study method			
Thyroidectomy	18	2.34 (1.97-2.76)	< 0.00001
FNA	6	1.27 (0.77-2.11)	< 0.0001
Selective FNA	3	2.37 (0.51-11.11)	0.002
Geographic area			
Europe	11	1.56 (1.01-2.40)	< 0.00001
Asia	9	2.79 (2.15-3.61)	< 0.00001
America	7	1.92 (1.56-2.35)	0.07
Publication year			
≥ 2010	20	2.32 (1.94-2.78)	< 0.00001
< 2010	7	1.65 (1.06-2.55)	< 0.00001
No. of PTCs			
≥ 100	18	2.12 (1.75-2.56)	< 0.00001
< 100	9	1.98 (1.16-3.37)	0.003
Tumor subtype			
PTC	24	2.06 (1.73-2.47)	< 0.00001
TC	3	2.64 (0.66-10.50)	0.002
Quality score			
≥ 7	2	4.79 (0.49-46.92)	0.11
< 7	22	2.06 (1.73-2.44)	< 0.00001
Adjusted for multivariate			
Yes	6	2.33 (1.32-4.14)	< 0.00001
No	21	1.88 (1.51-2.34)	< 0.00001
Mean age (year)			
≥ 48	15	1.98 (1.54-2.53)	< 0.00001
< 48	7	1.94 (1.41-2.67)	0.004
Sex ratio (female:male)			
≥ 5	10	2.17 (1.41-3.35)	< 0.00001
< 5	12	1.99 (1.61-2.45)	< 0.00001
Mean nodule size (cm)			
≥ 1.3	5	2.09 (1.64-2.66)	0.005
< 1.3	4	3.38 (2.56-4.46)	0.16

No., number; OR, odds ratio; CI, confidence interval; FNA, fine-needle aspiration; PTC, papillary thyroid carcinoma; TC, thyroid cancer.

### Sensitivity analysis and publication bias

**Figure 5:**
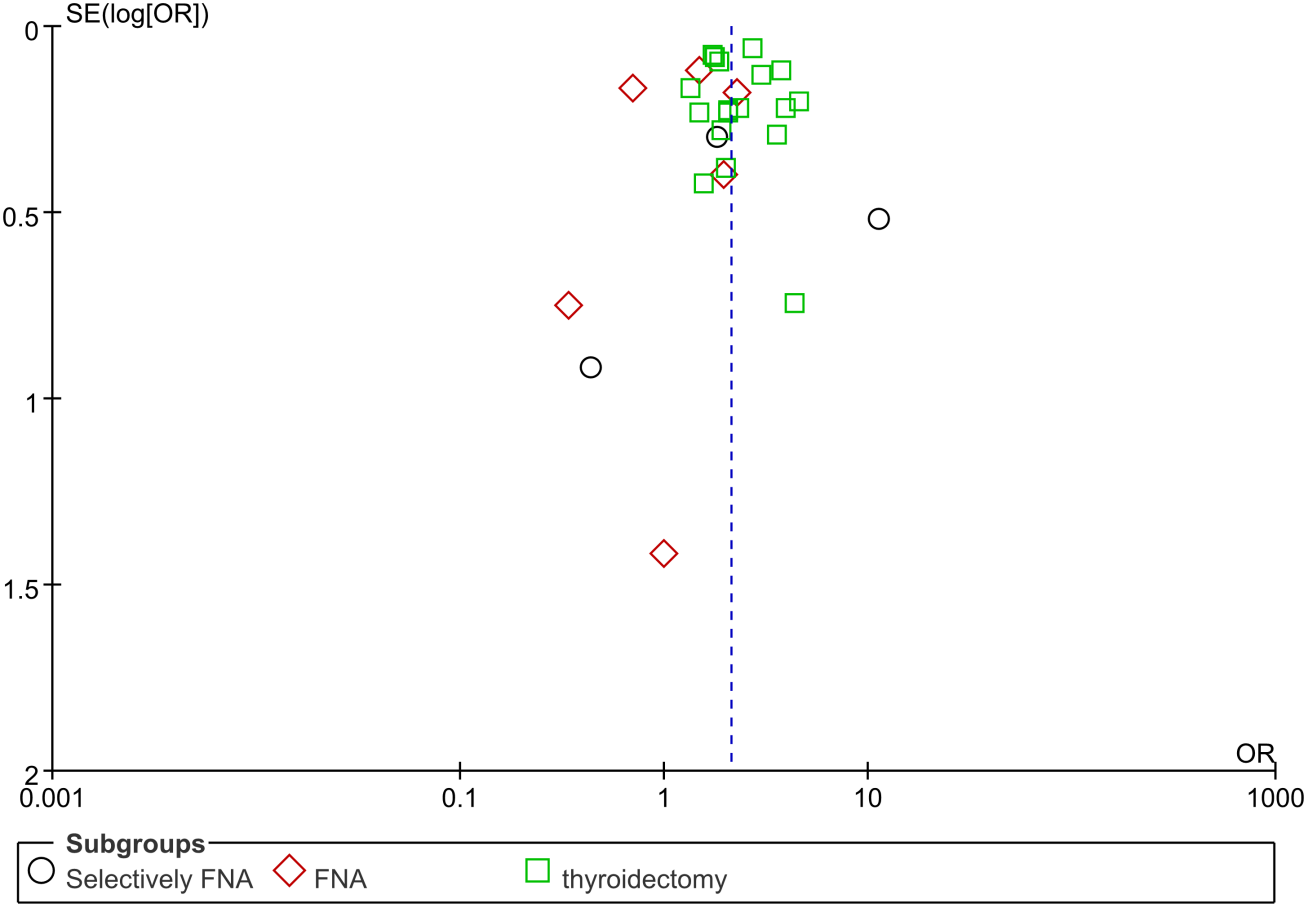
Funnel plot for publication bias in studies comparing the rate of PTC between HT and non-HT PTC, papillary thyroid carcinoma; HT, Hashimoto’s thyroiditis; FNA, fine-needle aspiration. Note: The black circles indicate selective FNA studies, the red rhombuses indicate FNA studies, and the green squares indicate thyroidectomy studies.

The sensitivity analyses revealed that no study had a significant influence on the overall estimates. The pooled ORs for HT varied from 2.04 (when excluding Siriweera et al. [18]) to 2.24 (when excluding Matesa-Anic et al. [23]). The shape of the funnel plot of studies assessing the association between HT and PTC risk seemed to be symmetrical, indicating the absence of publication bias (Figure 5).

## DISCUSSION

This meta-analysis showed that PTC was more often found in patients with HT than in patients without HT. However, there was significant heterogeneity among the studies, and the potential explanations may include variations in study methods, different patient inclusion criteria, and heterogeneous diagnostic criteria for HT and PTC. A previous meta-analysis found similar heterogeneity (Q = 39.664, df = 10, P < 0.001) [24].

In this meta-analysis, 3 types of studies were analyzed, namely, 3 selective FNA studies, 6 FNA studies, and 18 thyroidectomy studies. In the selective FNA studies, only selected patients underwent FNA or thyroidectomy; therefore, the rate of PTC among patients with HT (1.12%) was lower than that in the FNA studies (3.58%) and thyroidectomy studies (33.28%). However, the OR of PTC risk for HT (HT versus non-HT) (2.37) was not lower than that in the FNA studies (1.27) and thyroidectomy studies (2.34).

In the 3 selective FNA studies, one study reported a negative association between HT and thyroid carcinoma. Isik et al. compared the prevalence and sonographic features of thyroid carcinoma in 300 HT and 200 Graves’ disease (GD) patients [22]. In their study, all patients underwent ultrasonography, but only 32 HT and 55 GD patients underwent total thyroidectomy. No patient underwent FNA. The indications for surgery included patient preference, compressive symptoms, severe ophthalmopathy, patients whose ultrasonography revealed features that needed investigation, large goiter, and failure and/or serious side effects of antithyroid drugs, including agranulocytosis and hepatotoxicity. Two and three cases of thyroid carcinoma were confirmed in HT and GD patients, respectively, and the OR of thyroid carcinoma risk was 0.44 (95% CI: 0.07-2.66). In comparison, there were 19 and 52 cases of thyroid carcinoma in two other selective FNA studies [6, 16]. Mukasa et al. also compared the prevalence of thyroid carcinoma in 2,036 HT and 1,652 GD patients [16]. They performed ultrasound-guided FNA biopsy when the diameter of a nodule was >1 cm or when a nodule was suspected of being malignant base on the ultrasonographic features, including the presence of microcalcifications, nodule hypoechogenicity, irregular margins, and a shape taller than wide. Fifty-two patients who underwent surgery due to a suspicion of PTC were diagnosed with PTC. The study conducted by Chen et al. used claims data from the Taiwan National Health Insurance Program, and each patient was examined from the index date to the occurrence of cancer, death, or withdrawal from insurance or until the end of 2010 [6]. Therefore, there were significant differences in the patient population and surgery indication in these selective FNA studies, providing one possible explanation for the significant heterogeneity (I^2^ = 85%, P = 0.002).

There were also significant differences in the patient population of FNA studies. The inclusion criteria of FNA studies included consecutive patients [23, 28], patients with benign thyroid diseases [26], patients with cold thyroid nodules [8], patients with nodules ≥ 10 mm [21], and patients with nodules ≥ 5 mm and aged ≥ 18 years [4]. This may be one explanation for the wide incidence range of PTC, from 0.12% to 20.87%. Anil et al. prospectively compared the prevalence of thyroid carcinoma between 164 patients with HT and 551 patients without HT and found that thyroid nodules in patients with HT were no more likely to be malignant than those in patients without HT (OR = 0.35, 95% CI: 0.08-1.50). However, they included only patients with nodules ≥ 10 mm. In addition, only 4.9% of their HT group and 10% of the control group underwent thyroidectomy for thyroid nodules [21].

All 18 thyroidectomy studies in this meta-analysis showed that PTC is more often found in patients with HT than in patients without HT. In the clinical review conducted by Jankovic et al., all 8 thyroidectomy studies showed similar results [25]. However, Jankovic et al. believed that selection bias led to the statistically significant positive correlation. Lee et al. found that HT was more frequently observed in PTCs than in benign thyroid diseases (OR: 2.8; P < 0.001). In the funnel plots and Egger’s regression tests, there was no evidence of publication bias [24].

The relationship between inflammation and PTC is complex and still not completely understood [33]. Tamimi et al. found a significantly higher rate of lymphocytic infiltrate in patients with PTC, and the activated inflammatory response present in HT may create a favorable setting for malignant transformation [34]. The inflammatory response may cause DNA damage through the formation of reactive oxygen species, resulting in mutations that eventually lead to the development of PTC [25]. Both retrospective studies and a prospective study suggest that the association of HT with PTC is antibody-specific [4, 35, 36]. However, the mechanism underlying this association is not known. Paradoxically, the lymphocytic infiltrate of HT may be an immunological response with a cancer-retarding effect, contributing to a favorable outcome of PTC [37–39]. The relatively high prevalence of PTC in autopsy series may represent host immune control [5].

In conclusion, this meta-analysis includes the most comprehensive existing data on HT and PTC risk to date, with a large sample size, confirming that HT predisposed patients to the development of PTC. To better understand the potential mechanisms underlying this association, further studies are needed.

## MATERIALS AND METHODS

### Search strategy and inclusion criteria

We identified all relevant case-control and cohort studies published in English by searching two databases (PubMed and Embase) from the beginning of indexing to December 2015, using the following terms: (Hashimoto disease OR Hashimoto struma OR Hashimoto thyroiditis OR Hashimoto syndrome OR chronic lymphocytic thyroiditis) AND (thyroid tumor OR thyroid cancer OR thyroid carcinoma OR thyroid neoplasm) AND (cohort OR prospective OR case-control), following the Meta-analysis Of Observational Studies in Epidemiology (MOOSE) guidelines [40]. Two authors (XJ Lai and Y Xia) independently assessed and identified potentially relevant articles and reviewed the reference lists in the articles and associated reviews to identify additional studies. The inclusion criteria were as follows: (1) cohort study or case-control study published as an original article; (2) evaluation of the association of HT and thyroid cancer; and (3) availability ofthe OR and corresponding 95% CIs or sufficient information to enable their calculation. Abstracts or unpublished reports were not considered for inclusion in the meta-analysis.

### Data extraction and quality assessment

Two authors (XJ Lai and Y Xia) independently extracted the data from each original article. The extracted data included the first author, year of publication, country, study design, study method, period of enrollment, sample size (numbers of cases, controls, or non-cases or cohort size), and OR for HT and corresponding 95% CIs. Two authors (XJ Lai and Y Xia) independently assessed the quality of the included studies according to the Newcastle-Ottawa Scale (NOS) [41]. The NOS includes three broad perspectives: selection (four items), comparability (two items), and exposure/outcome (three items). The full score was 9 points, and a study with ≥ 7 points was defined as a high-quality study. Disagreements were discussed and resolved through consensus.

### Statistical analysis

The ORs were used for the meta-analyses. The pooled ORs with 95% CIs were calculated by the random-effects models for the association between HT and PTC [42]. Heterogeneity among articles was quantitatively assessed using the Q test and I^2^ statistic [43]. A significant heterogeneity was defined as I^2^ > 50% or Q-test reporting a P value < 0.1. To explore the potential sources of heterogeneity among studies, we conducted subgroup analyses for the strata of the study design, study method, geographic area, publication year, number of cases, tumor subtype, NOS quality score, whether adjusted for multivariate, mean age, sex ratio and mean nodule size. Sensitivity analyses were also performed by excluding one study at a time to clarify the influence of each study on the overall estimates. Publication bias was assessed by the funnel plot [44].

A P value < 0.05 was considered to be statistically significant. All P values were two-tailed. Statistical analyses were performed using RevMan 5 (www.cochrane.org) or the SPSS 11.5 software package (SPSS, Chicago, IL).

## Abbreviations

HT: Hashimoto’s thyroiditis
PTC: papillary thyroid carcinoma
FNA: fine needle aspiration
OR: odds ratio
CI: confidence interval
GD: Graves’ disease
NOS: Newcastle-Ottawa Scale
